# Smoking prevalence, perceptions and cessation practices among dental hygienists in Japan: A cross-sectional study

**DOI:** 10.18332/tid/226401

**Published:** 2026-09-29

**Authors:** Chika Watanabe, Koji Inagaki, Yukie Watanabe, Mari Niino, Izumi Kanamori, Mieko Kawano, Mayumi Shimokawa, Toshimi Kosaka, Junko Inukai

**Affiliations:** 1 Department of Dental Hygiene, Aichi Gakuin University Junior College, Nagoya, Japan; 2 Comprehensive Oral Health Research Institute, Nagoya, Japan; 3 Department of Periodontology, School of Dentistry, Aichi Gakuin University, Nagoya, Japan; 4 Aichi Gakuin University Junior College, Dental Hygienist Recurrent Training Center, Nagoya, Japan; 5 Dental Hygienists Association of Aichi Prefecture, Nagoya, Japan; 6 Dental Hygienists Association of Tokushima Prefecture, Tokushima, Japan; 7 Dental Hygienists Association of Kagoshima Prefecture, Kagoshima, Japan

**Keywords:** smoking cessation, dental hygienists, smoking, tobacco

## Abstract

**INTRODUCTION:**

Smoking is a risk factor for oral diseases such as periodontal disease and oral cancer. Dental hygienists play an important role in oral health promotion and smoking cessation support. The aim of this study was to clarify the smoking status, tobacco perceptions, and smoking cessation support among dental hygienists (DHs) in Japan.

**METHODS:**

A cross-sectional study using an anonymous self-administered questionnaire survey was conducted among 1481 DHs from June to July 2024. The survey items included smoking status, secondhand smoke exposure, Kano test for social nicotine dependence (KTSND) score, and implementation of smoking cessation support by DHs engaged in clinical practice.

**RESULTS:**

Valid responses were obtained from 648 (43.8%) participants; after excluding six men, 642 female DHs (43.3%) remained for analysis. Among them: 575; 10; and 57 were never, current, and former smokers, respectively, with a smoking prevalence of 1.6%. Secondhand smoke exposure was reported by 189 (29.4%) participants. The median KTSND score was 13.0, with 188 participants (29.3%) scoring ≤9 points, thereby meeting the criteria for having a correct perception of smoking. KTSND scores were significantly lower among never smokers (12.0) than among former (15.0) and current smokers (19.0) (both p<0.01). The rate at which smoking cessation support for patients with periodontal disease was implemented by DHs in clinical practice was 51.9%.

**CONCLUSIONS:**

The study findings suggest that although smoking prevalence among female DHs in Japan is low, misconceptions about tobacco remain prevalent. Enhancing tobacco-related education and training for DHs is essential to strengthen their ability to provide effective smoking cessation support and promote oral health.

## INTRODUCTION

The smoking rate among adult women in Japan has gradually declined, decreasing from 9.0% in 2012 to 6.5% in 2024^1^. However, heated tobacco products (HTPs) have become increasingly prevalent since 2015^2-4^. Specifically, among female smokers in 2024, the proportions of those using cigarettes, HTPs, both (dual users), and other products were 55.2%, 39.3%, 4.8%, and 0.6%, respectively, highlighting the emergence of dual users alongside exclusive HTP users^1^. Furthermore, HTP use among female smokers in their 20s increased from 52.9% in 2019 to 64.0% in 2024, indicating rapid expansion in this age group^1,5^. In addition to cigarettes and HTPs, other products such as electronic cigarettes, smokeless tobacco, and waterpipes (hookahs) are available in Japan. However, their usage remains largely unquantified^2,6^.

Nicotine dependence is classified as a disease, and it can make smoking cessation difficult, resulting in continued tobacco use. Smoking is a major risk factor for oral diseases such as periodontal disease and oral cancer (Level 1: sufficient scientific evidence to infer a causal relationship, based on the classification system adapted from the U.S. Surgeon General’s Report)^2^. Moreover, individuals exposed to secondhand smoke face similar health risks^2^. Consequently, in 2006, the Japan Dental Hygienists’ Association prioritized the promotion of smoking cessation among dental hygienists (DHs) as the first item in its declaration of smoking cessation^7^. Accordingly, tobacco-related education for DHs may be an important component of future smoking cessation support activities. Nevertheless, to the best of our knowledge, studies on the smoking and secondhand smoke exposure status of DHs, including their use of HTPs and perceptions of tobacco use, are lacking^8-11^.

Therefore, the aim of this study was to clarify the smoking status, tobacco perceptions, and smoking cessation support among DHs in Japan.

## METHODS

### Study design and participants

This cross-sectional study using an anonymous self-administered questionnaire on smoking awareness^12,13^ was conducted from June to July 2024. A total of 1481 DHs were recruited using self-selection sampling with the cooperation of the Aichi, Kagoshima, and Tokushima Prefectural Dental Hygienist Associations, Aichi Gakuin University Junior College Dental Hygienist Recurrent Training Center, and DH Training School. An anonymous self-administered questionnaire was distributed by email to all members of these organizations. DHs belonging to the participating organizations were considered eligible, with no restrictions on sex, age, years of professional experience, and clinical practice status. This study was reviewed and approved by the Ethics Committee of Aichi Gakuin University Junior College (Approval number: 24-006). The study protocol adhered to the guidelines of the Declaration of Helsinki.

### Sample size calculation

The 2024 National Health and Nutrition Survey revealed a smoking rate of 6.5% among women, nearly 44.1% of whom were HTP users^1^. Therefore, assuming a smoking rate of 10% with a confidence level of 95%, the required sample size for this study was 554. The free statistical software EZR was used for sample-size calculation^14^.

### Survey items

The survey items included age, sex, affiliation, smoking status (cigarettes, HTPs, e-cigarettes, smokeless tobacco, and waterpipes), smoking cessation measures at work, secondhand smoke exposure, Kano test for social nicotine dependence (KTSND) score, perceptions of HTPs, and history of visiting establishments that serve waterpipes. For smokers, additional items included past cessation attempts and the stage of behavioral change regarding cessation. For DHs engaged in clinical practice, views on periodontal treatment for smokers, frequency of smoking cessation support, and reasons for not providing such support were additionally assessed. Moreover, although nicotine-containing and non-nicotine versions of e-cigarettes and waterpipes are distributed, details regarding the nicotine content in these devices were not asked.

According to KTSND, social nicotine dependence is defined as ‘a psychological state in which smoking is perceived as an act rooted in society, with cultural significance, by glorifying, justifying, and rationalizing smoking while denying its harms’^15,16^. KTSND comprises 10 questions scored on a 4-point Likert scale (0–3 points each), with a total possible score of 30. Individuals with a score of ≤9 are classified as having a correct perception of smoking^15,16^. Furthermore, the KTSND quantifies three factors: ‘glorification of smoking (assertion of taste and culture)’, ‘rationalization/justification of smoking (overestimation of benefits)’, and ‘denial of the harms of smoking and secondhand smoke’. This scale is applied for evaluating not only current smokers but also never smokers, former smokers, and children, and is suitable for longitudinal evaluation^8,17^.

Regarding perceptions of HTPs, responses reflecting incorrect perceptions (‘Yes’ or ‘Unsure’) were assigned 1 point, whereas responses reflecting a correct perception (‘No’) were assigned 0 points. The total score (maximum 10 points) was calculated, with higher scores indicating a greater degree of misconception about HTPs^12,13,18^.

### Definitions of cigarette smoking and/or HTP use

According to their smoking status, the participants were categorized as non-smokers, experimental smokers, never smokers, current smokers, and former smokers. Non-smokers were individuals who had never smoked or used tobacco products in their lifetime. Experimental smokers were individuals who smoked at least once but had a lifetime cumulative consumption of <100 cigarettes^19^. Never smokers were a combined category including ‘never smokers’ and ‘experimental smokers’, defined as individuals without a history of habitual smoking. Current smokers were individuals who reported habitual smoking at the time of the survey. Former smokers were individuals with a history of habitual smoking who reported that they were not smoking at the time of the survey. Participants were further classified into the following two groups based on the type of tobacco product used: exclusive users, defined as individuals who used only one type of tobacco product (such as combustible cigarettes or HTP), and dual or multiple users, defined as individuals who concurrently used two or more types of tobacco products.

### Data collection

Data were collected using an anonymous self-administered web-based questionnaire. Participants were provided with a written explanation of the study objectives and procedures, and completion of the questionnaire was considered to indicate informed consent to participate. Participants were informed of their right to withdraw from the study at any time without penalty. All collected data were anonymized and handled with strict confidentiality.

### Statistical analysis

Descriptive statistics were used to summarize participant characteristics. Categorical variables are presented as frequencies and percentages, while continuous variables are presented as means ± standard deviations or medians with interquartile ranges, as appropriate. The normality of KTSND scores was assessed using the Shapiro–Wilk test. Because KTSND scores were not normally distributed, nonparametric tests were applied. KTSND scores were compared between groups using the Mann–Whitney U test, and differences among smoking status categories were assessed using the Kruskal–Wallis test. Factors associated with the implementation of smoking cessation support were analyzed using the chi-squared test. Statistical analyses were performed using the Statistical Package for Social Sciences, version 29 (IBM Corp., Armonk, NY, USA)^20^. A p<0.05 was considered statistically significant. This manuscript adheres to the Strengthening the Reporting of Observational Studies in Epidemiology guidelines for cross-sectional studies^21^.

## RESULTS

### Sample characteristics

A total of 653 responses (response rate 44.1%) were obtained. Of these, 648 (43.8%) were considered valid respondents, with no missing data regarding age or the recognition items for the four types of tobacco products other than cigarettes. Because the number of male respondents and respondents who preferred not to disclose their sex was small (n=6; three men and three who preferred not to disclose their sex), these individuals were excluded from the analysis. The final sample included 642 female DHs (43.3%). Thus, the required sample size was achieved. The mean age of the participants was 45.7 ± 13.1 years (range: 21–76 years). Among the participants, 578 (90.1%) were never smokers, 60 (9.3%) were former smokers, and 4 (0.6%) were current smokers (Table 1).

**Table 1:** Characteristics of the study participants (N=642)

*Characteristics*	*Data* *n (%)*
**Age** (years), mean ± SD (range)	45.7 ± 13.1 (21–76)
20–29	98 (15.3)
30–39	109 (17.0)
40–49	159 (24.8)
50–59	181 (28.2)
60–69	84 (13.1)
≥70	11 (1.6)
Overall smoking status*	
Never	575 (89.5)
Former	57 (8.9)
Current	10 (1.6)
**Cigarette smoking status**	
Never	578 (90.1)
Former	60 (9.3)
Current	4 (0.6)
**HTP using status**	
Never	628 (97.8)
Former	8 (1.2)
Current	6 (0.9)
**Secondhand smoke exposure**	
Yes	189 (29.4)
No	453 (70.6)
Smoking cessation measures at workplace	
Non-smoking premises	357 (55.6)
Indoor smoking	139 (21.7)
Indoor smoking areas	25 (3.9)
No regulations	5 (0.7)
Unknown	82 (12.8)
Others	34 (5.3)
**Engaged in clinical practice**	
Yes	326 (50.8)
No	316 (49.2)

* Overall smoking status was defined based on the use of any of the five tobacco product types available in Japan (cigarettes, HTP, e-cigarettes, smokeless tobacco, and waterpipes).

### Smoking status of DHs

Ten participants (1.6%) were identified as current smokers (Table 2). Of these, seven (1.1%) and three (0.5%) were exclusive and dual or multiple users, respectively. Regarding specific products, cigarettes were used by four individuals (0.6%), two of whom were exclusive users and two were dual users who concurrently used HTPs. The overall prevalence of HTP use was 0.9% (six individuals). Regarding other products, one participant (0.2%) exclusively used e-cigarettes and another (0.1%) exclusively used waterpipes; no smokeless tobacco users were identified.

**Table 2:** Smoking status among female dental hygienists in Japan, findings from a 2024 cross-sectional survey (N=642)

*Smoking status*	*n*	*%*
Exclusive smokers	7	1.1
Dual or multiple users	3	0.5
Smokers of any of the five types of tobacco products	10	1.6
**Smoking by product**		
**Cigarettes**		
Never smoker*	578	90.1
Non-smoker	522	81.4
Experimenter	56	8.7
Former smoker	60	9.3
Smoker	4	0.6
**HTPs**		
Never smoker*	628	97.8
Non-smoker	615	95.8
Experimenter	13	2.0
Former smoker	8	1.2
Smoker	6	0.9
**E-cigarettes**		
Never smoker*	640	99.6
Non-smoker	640	99.6
Experimenter	0	0.0
Former smoker	1	0.2
Smoker	1	0.2
**Smokeless tobacco**		
Never smoker*	642	100.0
Non-smoker	641	99.8
Experimenter	1	0.2
Former smoker	0	0.0
Smoker	0	0.0
**Waterpipes**		
Never smoker*	641	99.9
Non-smoker	616	96.0
Experimenter	25	3.9
Former smoker	0	0.0
Smoker	1	0.1
**Total smokers** (including overlaps)	12	-

* Never smokers have been classified as non-smokers plus experimenters. HTPs: heated tobacco products. The total number of smokers represents the unique count after removing overlaps due to use of multiple products; therefore, it does not equal the sum of number of smokers of individual tobacco products.

Past cessation attempts were reported by three of the four cigarette users and four of the six HTP users. Analysis of the stage of behavioral change for smoking cessation revealed that among cigarette users, one (25.0%) was in the pre-contemplation stage and three (75.0%) were in the contemplation stage. With regard to HTPs, two users (33.3%) were in the pre-contemplation stage and four (66.7%) were in the contemplation stage. While both the e-cigarette and waterpipe users (100% each) remained in the pre-contemplation stage.

Secondhand smoke exposure was reported by 189 (29.4%) participants. Among the sources of exposure (multiple responses allowed), the most frequent were spouses or partners (119; 63.0%), followed by fathers (47; 24.9%), children (39; 20.6%), and mothers (17; 9.0%). Other sources included younger brothers (13; 6.9%), grandfathers (7; 3.7%), older brothers (7; 3.7%), older sisters (6; 3.2%), grandmothers (3; 1.6%), younger sisters (2; 1.1%), and fathers-in-law (1; 0.5%). Exposure from neighbors or unknown sources was reported by one participant (0.5%).

### Smoking cessation measures at work

Smoking cessation measures at workplaces were non-smoking premises (357; 55.6%), indoor smoking (139; 21.7%), indoor smoking areas (25; 3.9%), no regulations (5; 0.7%), unknown (82; 12.8%), and others (34; 5.3%).

### KTSND scores

The mean KTSND score for all participants was 12.2 ± 5.3 (range: 0–30), with a median of 13.0. A total of 188 participants (29.3%) scored ≤9 points, meeting the criteria for having a correct perception of smoking. The KTSND scores were significantly lower among never smokers (12.0) than among former (15.0, p<0.01) and current (19.0, p<0.01) smokers (Table 3).

**Table 3:** KTSND scores according to smoking and secondhand smoke exposure status among female dental hygienists in Japan, 2024 (N=642)

*Smoking status*	*n*	*KTSND score* *Median (IQR)*
Total	642	13.0 (8.0–16.0)
Never smoker	575	12.0 (8.0–16.0)^a,b^
Non-smoker	502	12.0 (8.0–16.0)
Experimenter	73	13.0 (8.5–15.0)
Former smoker	57	15.0 (12.0–18.0)^a^
Smoker	10	19.0 (13.8–21.0)^b^
Group not exposed to secondhand smoke	453	12.0 (8.0–16.0)
Group exposed to secondhand smoke	189	13.0 (9.0–17.0)

a Among DHs, the KTSND scores were significantly lower for non-smokers than for former smokers (Kruskal–Wallis test, p<0.01).

b Among DHs, the KTSND scores were significantly lower for non-smokers than for current smokers (Kruskal–Wallis test, p<0.01). KTSND: Kano test for social nicotine dependence. IQR: interquartile range.

### Perception scores for HTPs

The mean perception score for HTPs was 3.2 ± 2.9 (range: 0–10), with a median of 2.0. Only 109 participants (17.0%) correctly identified all 10 items. The percentages of correct responses for each item are listed in Table 4.

**Table 4:** Perceptions of heated tobacco products (HTPs) among female dental hygienists in Japan, responses to ten questionnaire items in a 2024 cross-sectional survey (N=642)

*Items*	*n*	*%*
Harmful to health	542	84.4
Harmful to others (secondhand effects)	512	79.8
Cannot be used in non-smoking areas	502	78.2
Is a form of smoking	494	76.9
Pollutes the air	470	73.2
Has an odor	469	73.1
Not effective as a smoking-cessation aid	393	61.2
Not a substitute for conventional cigarettes	198	30.8
Is addictive	514	80.1
Contains tobacco leaves	294	45.8

### History of visiting establishments serving waterpipes

Regarding a history of visiting cafés or establishments that serve waterpipes, 21 participants (3.3%) reported having visited once but discontinued immediately, one (0.2%) reported visiting for a period of time but had since stopped, and eight (1.2%) reported occasional visits.

### Current status of smoking cessation for patients among clinical DHs

Among the participants, 326 (50.8%) were engaged in clinical practice. Regarding the implementation of smoking cessation support for patients with periodontal disease, 15 (4.6%) provided it to all patients, 154 (47.3%) provided it when necessary, 111 (34.0%) had not yet implemented it, and 46 (14.1%) did not intend to provide smoking cessation; the overall implementation rate was 51.9%.

The primary reasons cited for not providing smoking cessation support (multiple responses allowed) included lack of instructions from a dentist (82; 48.5%), lack of time (74; 43.8%), lack of knowledge regarding support methods (68; 40.2%), lack of educational materials (59; 34.9%), difficulty in approaching patients about the topic (45; 26.6%), and lack of objective evaluation methods or equipment (29; 17.2%). Other reasons included ‘not covered by health insurance’ (18; 10.7%), ‘patient refusal’ (17; 10.1%), ‘the director of the clinic smokes’ (14; 8.3%), and ‘support is already being provided by a physician’ (8; 4.7%). A small number of respondents felt it was unnecessary in a dental setting or believed that smoking does not affect periodontal treatment outcomes.

### Factors associated with the implementation of smoking cessation support

A significant association was found between the implementation of support and the perception that smoking cessation is easier to promote in dental settings by demonstrating changes in the teeth and gingival color. Specifically, 143 participants (55.2%) who answered ‘Agree’ or ‘Somewhat agree’ were actively providing support; those who agreed were significantly more likely to provide support than those who answered ‘Disagree’, ‘Neutral’, or ‘Unsure’ (p<0.05) (Table 5).

**Table 5:** Factors associated with the implementation of smoking cessation support among female clinical dental hygienists in Japan, 2024 (N=326)

*Question*	*Response*	*Implemented*		*Not* *implemented*		*p*
		*n*	*%*	*n*	*%*	
**Do you think visualizing changes in the color of teeth and gingiva makes it easier to provide smoking-cessation support in dentistry?**	Agree/somewhat agree	143	55.2	116	44.8	0.02
	Disagree/neutral/unknown	26	38.8	41	61.2	
**Do you think there is a high prevalence of smokers among patients with severe periodontal disease?**	Agree/somewhat agree	144	56.9	109	43.1	<0.01
	No different from non-smokers/unknown (status unassessed)/unknown	25	34.2	48	65.8	

Implementation status was categorized as ‘Implemented’ (for all patients or as needed) or ‘Not implemented (not yet started or no intention to provide support) and analyzed using the chi-squared test.

Similarly, regarding the perception that severe periodontal disease is more prevalent among smokers, 144 participants (56.9%) answered ‘Agree’ or ‘Probably so’. Those who agreed with this observation were significantly more likely to implement smoking cessation support compared to those who responded, ‘No difference from never smokers’, ‘Unknown because smoking status is not assessed’, or ‘Unsure’ (p<0.01).

## DISCUSSION

### Smoking prevalence among female DHs

Among Japanese women, the smoking rate is the highest (10.5%)^1^ among those aged 40–49 years, which corresponds to the mean age in this study (45.7 years). In contrast, the smoking rate in this study was considerably lower (1.6%). The declaration on smoking cessation by the Japan Dental Hygienists’ Association^7^, and dental education for students aspiring to become DHs likely contributed to the low prevalence of smoking^22^. Compared with the previously reported rates (0.5% in 2019^9^, 0% in 2020^10^, and 2.3% in 2022^11^), the rate in this study was nearly equivalent to that in 2022. This increase relative to that in 2019 may be attributed to the larger number of tobacco product types included in the current survey.

### Perceptions and knowledge of HTP and alternative tobacco products

In this study, only 17.0% of the participants correctly identified all 10 HTP items. Notably, only 30.8% correctly identified HTPs as not being a substitute for cigarettes, and approximately 70% believed they were a substitute or were unsure. Although HTPs contain harmful substances such as nicotine, similar to cigarettes, marketing by HTP manufacturers emphasizes that harmful substances are reduced compared to those in cigarettes^2^. This may lead consumers to mistakenly perceive HTP as viable substitutes, potentially encouraging their use. Therefore, accurate education regarding smoking cessation is essential for DHs.

Regarding tobacco products other than cigarettes and HTPs, one participant each reported using e-cigarettes and waterpipes, whereas 30 participants had visited waterpipe cafés at least once. These findings may highlight the importance of maintaining awareness and education regarding alternative tobacco products. Furthermore, waterpipe cafés are rapidly becoming popular among young people^6^; thus, disseminating knowledge about the physiological effects of all tobacco products, including waterpipes, and discouraging visits to such establishments are important.

### Psychological aspects of smoking: social nicotine dependence

The median KTSND score in this study was 13.0, which was higher than those reported previously (12.0 in 2019^9^, 11.0 in 2020^10^, and 10.0 in 2022^11^). Additionally, the percentage of participants within the criteria range (≤9 points) was 29.3%, which was lower than that in 2019 (33.2%)^9^ and 2020 (37.9%)^10^. This discrepancy may arise from the fact that the study population included female DHs who received smoking cessation education during schooling and those who did not. Therefore, promoting an accurate understanding of smoking habits is necessary. Analysis of the KTSND scores according to the smoking status showed that scores among never smokers were significantly lower than those among current and former smokers. This suggests that current or past smoking experience is associated with a more tolerant perception of smoking.

### Implementation of smoking cessation and barriers in clinical practice

The rate of implementation of smoking cessation support was 51.9%. Compared to those in previous reports (50.9% in 2019^9^, 53.5% in 2020^10^, and 69.6% in 2022^11^), this rate was similar to that in 2019 and 2020 but lower than that in 2022. The higher rate in 2022 may be explained by the fact that the participants were DHs who were members of the Japanese Society of Periodontology. Therefore, their awareness of smoking cessation may have been higher owing to the strong correlation between smoking and the exacerbation of periodontal disease^2^. Factors associated with implementing support included knowledge of the relationship between severe periodontal disease and smoking and the perception that support is easier to provide by demonstrating changes in the teeth and gingival color.

Conversely, the most common reasons for not providing support were ‘lack of instructions from a dentist’ and ‘lack of time’. Additionally, 8.3% of respondents selected ‘the director of the clinic smokes’. As 26.3% of the workplaces included in the analysis allowed smoking, dentists and DHs must take the initiative for smoking cessation and promote policies for smoke-free premises^12,13^.

As DHs provide dental care under the instructions of dentists, few appear to proactively offer smoking cessation support alongside other treatments. The lack of knowledge regarding support methods is an additional inhibitory factor. Therefore, establishing an environment in which DHs can proactively provide support by acquiring knowledge through workshops on the relationship between smoking and oral diseases, including periodontal disease, and by utilizing educational materials and procedural manuals is necessary^23^.

### Limitations

The study has some limitations. First, the presence of nicotine in e-cigarettes and waterpipes was not investigated. Second, we could not confirm whether former users of HTPs and e-cigarettes had disposed of their devices; thus, the risk of relapse could not be excluded. Third, due to the small number of smokers, the large difference in the number of smokers and non-smokers may have affected the accuracy of the between-group comparison. Fourth, since the survey did not ask about the details of the workplace or the work status, those who are currently not working may have answered ‘other’ for smoking cessation measures at the workplace. Fifth, participants were recruited from selected dental hygienist associations and institutions rather than through random sampling; therefore, the representativeness and generalizability of the findings may be limited. Sixth, because this study used a cross-sectional design, causal relationships between smoking-related factors and smoking cessation support could not be inferred. Seventh, although the questionnaire was anonymously completed, self-reported data may have been subject to recall bias, misclassification bias, and social desirability bias, particularly with regard to smoking-related behaviors. Eighth, the response rate was relatively low (43.8%), and this may have introduced non-response bias and limited the representativeness of the study population. Finally, multivariable analyses to adjust for potential confounding factors were not performed; this may have limited the interpretation of the observed associations.

## CONCLUSIONS

The findings of this study suggest that although smoking prevalence among female DHs in Japan is low, gaps remain in tobacco-related knowledge and the implementation of smoking cessation support. These findings highlight the potential importance of strengthening tobacco-related education and maintaining awareness of emerging tobacco products among DHs. Enhancement of training programs on the relationship between smoking and oral health, provision of practical approaches for smoking cessation support, and development of educational materials may represent potential strategies to support DHs in providing effective smoking cessation guidance in dental settings.

## Data Availability

The data supporting this research are available from the authors on reasonable request.
